# University music club participation, belonging, identity development, and agency: an exploratory pretest-posttest study with attention to gender-marginalized students

**DOI:** 10.3389/fpsyg.2026.1846732

**Published:** 2026-06-04

**Authors:** ZiLong Guo, Anqian Wang, Keran Li, Gao Qi, Yaojue Xie

**Affiliations:** 1 Public Basic Department, Zhejiang Police College, Hangzhou, China; 2 Faculty of Educational and Liberal Studies, City University, Petaling Jaya, Selangor, Malaysia; 3 Faculty of Music and Dance, Liaocheng University, Liaocheng, China; 4 Global Convergence Studies Department, Kangwon National University, Chuncheon-si, Republic of Korea; 5 Music Academy Department, Dankook University, Yongin-si, Republic of Korea

**Keywords:** agency, belonging, expressive safety, gender-marginalized students, identity development, music education, recognition, university music clubs

## Abstract

This study examined whether participation in university music club activities was associated with students’ belonging, identity development, and agency, with exploratory attention to gender-marginalized students. Using a quantitatively dominant design supplemented by contextual qualitative materials, the study followed 288 undergraduate students from six universities and nine music-related student clubs across an 8-week activity cycle. A pretest-posttest survey design was employed, supplemented by brief activity feedback forms, short interviews, structured observations, and organizer logs. The results showed that belonging, identity development, agency, perceived recognition, and expressive safety all increased significantly over time, with small-to-moderate effect sizes. More frequent participation was positively associated with the three focal outcomes, while perceived recognition and expressive safety were also positively linked to students’ developmental and participatory experiences. Regression analyses indicated that identity development was most strongly associated with belonging and expressive safety, whereas agency was more directly associated with participation frequency and belonging. Bootstrapped indirect-effect analyses further suggested that perceived recognition and expressive safety may help explain associations between participation frequency and belonging, identity development, and agency, with expressive safety showing a particularly salient association with identity development. Supplementary analyses showed that gender-marginalized students reported consistently lower mean levels across the focal variables, although these differences did not reach statistical significance and should be interpreted as preliminary. Overall, the findings suggest that university music clubs may function as socially meaningful educational environments in which recognition, inclusion, and voice are supported through participation, while causal and subgroup claims should remain cautious.

## 1 Introduction

Educational spaces are not neutral. For gender-marginalized students, participation in school and university life often unfolds within environments structured by visibility, normative expectations, evaluation, and social recognition. Even when overt discrimination is absent, more subtle forms of marginalization may persist: being misunderstood, being rendered invisible, having one’s self-expression misread, or experiencing participation as conditional rather than fully legitimate. These forms of marginalization matter because they shape whether students feel that they belong, whether they can safely articulate who they are, and whether they experience themselves as active agents rather than peripheral participants. Research on school belonging has long shown that feeling accepted, respected, and supported is closely tied to educational adjustment and motivation (Goodenow, 1993), while work on LGBTQ+ youth has repeatedly documented that school victimization, exclusion, and unsupportive climates are associated with poorer belonging, safety, and wellbeing (Russell et al., 2011; Day et al., 2019). More recent evidence further suggests that gender- and sexuality-diverse students often report lower access to protective school assets and supportive school resources than their peers, underscoring the continuing importance of relational and contextual supports in educational settings (Coulter et al., 2021; Leung et al., 2022).

At the same time, existing scholarship leaves several important gaps. First, although research on gender diversity in education, school climate, and LGBTQ+ support has expanded, much of it remains focused on general inclusion, safety, or policy environments rather than on the specific interactional mechanisms through which students come to feel recognized, able to participate, and able to develop a coherent sense of self. Second, while studies of school supports such as inclusive policies, social support systems, and student-led alliances show that affirming environments can strengthen school functioning, mental health, and belonging, these findings have concentrated mainly on K–12 school climate and GSA-type structures rather than on music education or co-curricular artistic settings (Baams and Russell, 2021; Poteat et al., 2024; Poteat et al., 2025). Third, music and arts education research has often emphasized creativity, emotional expression, and participation, but has less often centered gender-marginalized students as a focal group or examined belonging, identity development, and agency within a single explanatory framework. Finally, there remains a need for research designs that move beyond static association and instead assess whether sustained participation in a structured educational music context is linked to meaningful change over time.

Music-based educational settings may be especially important for addressing these gaps because music offers mechanisms that differ from many conventional academic spaces. First, music creates a multimodal channel of expression: students can communicate through sound, rhythm, lyrics, improvisation, performance, and collaborative creation, thereby reducing dependence on a single verbal mode of self-presentation and making room for alternative forms of expression and self-definition (North and Hargreaves, 1999; Saarikallio et al., 2020). Second, music-making can facilitate a reorganization of relationships: ensemble practice, rehearsal, and joint music-making require listening, coordination, responsiveness, and mutual adjustment, which can redistribute who is heard, who is recognized, and how participation is valued within a group (Kirschner and Tomasello, 2010; Stolp et al., 2022a,b). Third, music can function as a space for identity rehearsal and agency development. Prior work in music education has suggested that musical engagement can support self-construction, agency, and the negotiation of personal meaning, especially when learners are offered psychologically safe environments in which experimentation and participation are possible (Karlsen, 2011; Hendricks et al., 2014; Stolp et al., 2022a,b; Sutela et al., 2020). Taken together, these studies suggest that music is not merely a therapeutic or motivational add-on; rather, it may operate as a relational and expressive medium through which belonging, identity, and agency are jointly shaped.

Against this background, the present study examines participation in university music club activities as a meaningful educational context for understanding how students, including gender-marginalized students, may experience belonging, identity development, and agency. The study makes three more modest contributions. First, it extends existing work on belonging, recognition, identity, and student voice into a co-curricular music setting that has received comparatively limited empirical attention. Second, it offers an exploratory relational-developmental framework linking participation in music club activities to belonging, identity development, and agency through perceived recognition and expressive safety. Third, it uses a quantitatively dominant pretest-posttest design, supplemented by contextual qualitative materials, to examine whether structured participation in music-based educational communities is associated with positive change across an 8-week activity cycle. In doing so, the study positions university music clubs not as inherently transformative spaces, but as potentially meaningful social environments in which recognition, voice, and self-development may be negotiated in ways that are especially relevant for students whose identities are often marginalized in mainstream educational life.

## 2 Literature review

### 2.1 Belongingness and school belonging in educational contexts

Belongingness provides one of the most important theoretical foundations for understanding why educational environments matter for student development. At the broadest level, belonging has been conceptualized as a fundamental human motivation rooted in the need to form and maintain stable, positive interpersonal bonds (Baumeister and Leary, 1995). Within educational settings, this general need takes a more specific form as school belonging, namely the extent to which students feel accepted, respected, included, and supported within a learning community. In this sense, belonging is not simply an emotional preference or a by-product of successful adjustment, but a relational condition that shapes whether students experience themselves as legitimate members of a collective. Osterman (2000) argued that the need to belong is central to students’ willingness to participate, engage, and invest themselves in school life, while later reviews and meta-analyses have consistently shown that school belonging is associated with a wide range of positive outcomes, including stronger engagement, better socio-emotional adjustment, and more favorable academic functioning (Allen et al., 2018; Korpershoek et al., 2020; Slaten et al., 2016). This suggests that belonging should be treated not as a peripheral affective variable, but as a central explanatory construct linking social environments to broader developmental processes. At the same time, scholarship has also demonstrated that belonging is socially produced rather than individually possessed. It emerges through repeated interactional signals that communicate whether one is welcomed, recognized, and entitled to participate. Research has shown that teacher–student relationships, peer relationships, and family involvement all contribute to adolescents’ sense of school belonging (Uslu and Gizir, 2017), and that belonging remains developmentally significant even in late adolescence and higher education contexts (Pittman and Richmond, 2007). Moreover, work on marginalized student populations has highlighted that belonging may be conditional, uneven, or fragile when students are formally included in educational spaces but do not feel genuinely seen or affirmed within them (Booker, 2006).

This theoretical perspective is especially relevant for the present study because gender-marginalized students may participate in educational communities while still encountering subtler forms of invisibility, misrecognition, or constrained participation. From a belongingness perspective, the issue is not only whether students are physically present in a setting, but whether they perceive themselves as legitimate and valued members of it. Applied to university music club participation, belonging theory suggests that such settings may matter not merely because they provide opportunities for artistic practice, but because they can function as relational communities in which members become visible to one another through repeated rehearsal, collaboration, and shared performance preparation. Music club activities create recurring occasions for mutual acknowledgment, role confirmation, and social inclusion, all of which may strengthen students’ sense of being “part of” a meaningful collective. In turn, stronger belonging may increase willingness to participate, willingness to express oneself, and willingness to take interpersonal and creative risks. This is especially important for students whose identities are more likely to be marginalized in mainstream educational life, because belonging may serve as the relational foundation that makes further developmental processes possible. For this reason, the present study treats belonging as a key mechanism rather than a background emotion: participation in music club activities is expected to foster experiences of inclusion and recognition, these experiences are expected to strengthen belonging, and stronger belonging is, in turn, expected to provide the social basis for subsequent identity development and agency.

### 2.2 Identity development and recognition in marginalized educational experience

Identity development provides a second essential theoretical foundation for the present study because it helps explain why being seen, acknowledged, or misrecognized in educational settings matters so deeply for young people’s self-formation. Developmental scholarship has consistently identified adolescence and emerging adulthood as crucial periods for constructing a coherent sense of self, not only through internal reflection but also through interaction with social contexts, transitions, and relationships. In this view, identity is not formed in isolation; rather, it develops through cycles of exploration, commitment, and self-interpretation as individuals attempt to integrate personal experience into a more stable understanding of who they are. Process-oriented identity research further suggests that identity development depends on opportunities to explore, test, and consolidate personally meaningful commitments, while research on self-concept clarity indicates that a more coherent and stable sense of self is associated with healthier functioning and stronger self-understanding (Campbell et al., 1996; Crocetti, 2017; Meeus, 2011). From this perspective, identity development is not merely a matter of claiming an identity, but of forming a sense of self that is coherent, meaningful, and sustainable over time.

Recognition theory strengthens this developmental perspective by highlighting that identity formation is inherently relational. Educational settings do not merely provide knowledge or skill training; they also communicate whether students are intelligible, valued, and legitimate as participants. Thus, being recognized by others is not a superficial interpersonal experience, but a condition that shapes whether young people can stabilize their self-understanding and express it with confidence. Conversely, misrecognition may interrupt this process by signaling that one’s self-presentation, way of participating, or claimed place in the community is not fully affirmed. This insight is especially important for gender-marginalized students, whose experiences may include subtle invalidation, constrained self-expression, or the need to negotiate visibility carefully. In university music clubs, these dynamics may be particularly salient because rehearsals, performances, and collaborative creation offer repeated opportunities not only for artistic practice but also for social acknowledgment and identity experimentation. Music-based participation may therefore support identity development when students experience the group as a place where their contributions are recognized, their self-expression is treated as legitimate, and their presence is not merely tolerated but affirmed. In the logic of the present study, identity development is thus treated as a relationally mediated process: participation in music club activities is expected to foster belonging and recognition, and these experiences, in turn, are expected to support stronger identity development and lay the groundwork for greater agency (Branje, 2022; Verhoeven et al., 2019).

### 2.3 An integrated relational-developmental framework

Taken together, belongingness theory, identity development/recognition theory, and agency theory can be integrated into a coherent relational-developmental framework for the present study. Belongingness theory suggests that students’ participation in educational communities depends not only on formal access, but also on whether they experience themselves as accepted, respected, and legitimately included within the group (Baumeister and Leary, 1995; Osterman, 2000). Identity development research further indicates that a stable and coherent sense of self is formed through socially situated processes of exploration, commitment, and self-interpretation, rather than through purely internal reflection (Crocetti, 2017; Meeus, 2011). At the same time, recognition-oriented perspectives emphasize that identity is strengthened or destabilized through interactional experiences of acknowledgment, affirmation, and misrecognition in institutional settings (Verhoeven et al., 2019). Agency theory adds a further layer by showing that meaningful participation requires more than presence or adjustment: students must also experience themselves as capable of expressing ideas, contributing to collective processes, and shaping what happens around them (Stenalt and Lassesen, 2022). Considered together, these perspectives imply that music-based participation may matter developmentally not simply because it offers opportunities for artistic engagement, but because it may reshape how students are socially received, how securely they experience themselves within the group, and how confidently they act within that environment.

This integrated perspective is especially relevant in the context of university music club activities, where rehearsal, collaborative preparation, shared performance, and feedback create repeated opportunities for students to become visible to one another in ways that are both interpersonal and expressive. Previous work in music and education suggests that musical participation can support both self-expression and agency when learners are given meaningful opportunities to contribute and when their contributions are treated as legitimate within the collective setting (Stolp et al., 2022a,b). From this standpoint, music club participation may first alter the relational conditions under which students experience themselves in the group—particularly whether they feel recognized, heard, and included. These relational experiences are likely to matter because they provide the interpersonal grounding of belonging; belonging, in turn, may offer the security necessary for students to express, test, and consolidate a more coherent sense of self; and a more coherent, affirmed sense of self may make active participation, contribution, and influence more likely. In this way, the present study does not treat belonging, identity development, and agency as isolated outcomes, but as theoretically connected dimensions of a broader developmental process. This perspective is particularly important for gender-marginalized students, whose participation in educational spaces may be shaped less by formal access alone than by whether the environment enables recognition, affirmation, and credible opportunities for voice.

### 2.4 Hypotheses

Building on the theoretical framework outlined above, the present study proposes that participation in university music club activities is associated with students’ belonging, identity development, and agency through relational and expressive conditions. Specifically, repeated participation in music-based activities is expected to be positively related to students’ experiences of recognition and expressive safety, which may in turn be associated with stronger belonging, identity development, and agency. On this basis, the following hypotheses were formulated.

*H1*: Participation in university music club activities will be associated with significant increases in belonging, identity development, agency, perceived recognition, and expressive safety over time.

*H2*: Greater involvement in music club activities, particularly in terms of participation frequency and duration, will be positively associated with students’ sense of belonging.

*H3*: Perceived recognition and expressive safety will be positively associated with belonging, identity development, and agency.

*H4*: Belonging and expressive safety will be positively associated with identity development.

*H5*: Belonging and more frequent participation in music club activities will be positively associated with agency.

*H6*: Perceived recognition and expressive safety will show significant indirect associations between participation frequency and the focal outcomes of belonging, identity development, and agency.

## 3 Methodology

### 3.1 Research design

This study employed a quantitatively dominant design supplemented by qualitative contextual materials to examine whether participation in university music club activities was associated with students’ belonging, identity development, and agency, with exploratory attention to gender-marginalized participants. The quantitative component served as the primary basis for assessing changes in the focal constructs across the activity cycle, whereas the qualitative materials were incorporated to provide contextual insight into how participants and organizers described the social conditions of club participation. Rather than conceptualizing music clubs merely as recreational extracurricular spaces, the present study treated them as socially meaningful settings in which students engage in repeated interaction, collaborative practice, performance preparation, and group-based meaning-making. In this sense, music club participation was understood as a sustained participatory context rather than as attendance at isolated optional activities. A pretest-posttest design was adopted. Given the voluntary nature of university club participation and the practical impossibility of random assignment within naturally occurring student organizations, the study did not attempt to construct a randomized experimental design or to establish definitive causal effects. Instead, it followed students across an 8-week activity cycle in real music club contexts, measuring the key variables before and after participation. This design was selected to preserve ecological validity while enabling a structured assessment of change over time. The supplementary qualitative materials were used in a complementary, background role rather than as an equal-weight qualitative strand. Quantitative survey data were used to examine whether students’ levels of belonging, identity development, and agency changed over the course of participation, whereas short interviews, brief feedback forms, structured observations, and organizer logs were used to contextualize how specific interactional features of music club activities may have supported or constrained such change. To improve interpretive credibility, several procedural controls were built into the study. First, all participants were recruited from comparable university music clubs operating within a similar time frame. Second, the same survey instruments were administered at pretest and posttest. Third, background variables such as age, year level, prior musical experience, duration of club participation, and weekly participation frequency were included in the analysis as controls. Fourth, multiple forms of supplementary contextual data were collected to help interpret the quantitative patterns.

### 3.2 Research setting and participants

The study was conducted in university music club settings, which served as the primary research context. These settings included student-led and semi-structured musical communities in which participants regularly engaged in rehearsals, ensemble practice, collaborative arrangement, performance preparation, and post-activity interaction. Compared with formal classroom teaching, university music clubs are characterized by voluntary participation, peer-based coordination, and varying degrees of public expression, making them particularly suitable for examining how students may move from relative invisibility to more active voice within a collective setting. Participants were recruited from six universities and nine music-related student clubs, including choir-based organizations, instrumental ensembles, popular music clubs, and broader music performance groups. Eligibility criteria were as follows: participants had to be currently enrolled undergraduate students, be active or newly registered members of a music club during the study period, and be willing to complete both waves of the questionnaire during one full cycle of club activities. Initial recruitment and research briefing reached 312 students. Of these, 298 participants completed the pretest survey, and 288 participants completed the posttest survey and provided usable matched responses, which constituted the final analytic sample. Participants ranged in age from 18 to 24 years, with a mean age of 20.20 years (SD = 1.24). Descriptive characteristics of the sample are presented in Tables 1, 2. In terms of gender, 168 participants (58.3%) identified as women, 93 (32.3%) identified as men, and 27 (9.4%) identified as gender-marginalized. In terms of year level, 97 students (33.7%) were second-year students, 82 (28.5%) were first-year students, 71 (24.7%) were third-year students, and 38 (13.2%) were fourth-year students. Regarding disciplinary background, 112 students (38.9%) were from humanities and social sciences, 96 (33.3%) were from science and engineering, 54 (18.8%) were from arts-related fields, and 26 (9.0%) were from other or interdisciplinary programs.

**Table 1:** Demographic characteristics of participants.

Variable	Category	*n*	%
Gender	Women	168	58.3
Men	93	32.3	
Gender-marginalized	27	9.4	
Year level	Second year	97	33.7
First year	82	28.5	
Third year	71	24.7	
Fourth year	38	13.2	
Disciplinary background	Humanities and social sciences	112	38.9
Science and engineering	96	33.3	
Arts-related fields	54	18.8	
Other / interdisciplinary programs	26	9.0	

Age: M = 20.20, SD = 1.24, range = 18–24.

**Table 2:** Music club participation characteristics of participants.

Variable	Category	*n*	%
Duration of club participation	6–12 months	79	27.4
3–6 months	73	25.3	
More than 1 year	72	25.0	
Less than 3 months	64	22.2	
Weekly participation frequency	Twice per week	126	43.8
Once per week	91	31.6	
Three times or more per week	71	24.7	
Prior formal musical experience	None	89	30.9
Less than 2 years	74	25.7	
2–5 years	69	24.0	
More than 5 years	56	19.4	

Gender-marginalized participants included students who identified as non-binary, genderqueer, transgender, self-described, or preferred not to be categorized within the gender binary.

Participants also varied in their club involvement and musical background. Specifically, 79 students (27.4%) had participated in their club for 6–12 months, 73 (25.3%) for 3–6 months, 72 (25.0%) for more than 1 year, and 64 (22.2%) for less than 3 months. In terms of weekly participation frequency, 126 students (43.8%) reported attending club activities twice per week, 91 (31.6%) once per week, and 71 (24.7%) three times or more per week. Regarding prior formal musical experience, 89 participants (30.9%) reported none, 74 (25.7%) reported less than 2 years, 69 (24.0%) reported 2–5 years, and 56 (19.4%) reported more than 5 years of formal musical experience. The subgroup of gender-marginalized participants constituted the focal subgroup in the present study. To protect participant autonomy and reduce the risk of forced categorization, gender identity was measured using an inclusive and optional self-report item that allowed participants to select, self-describe, or skip the question. All participants took part in the study on a voluntary basis. The study information sheet and questionnaire introduction clearly stated that participation was entirely optional, that any item could be skipped, and that withdrawal at any stage would not affect club membership, participation opportunities, or peer standing within the organization.

### 3.3 Operationalization of music club participation

In the present study, the focal form of participation was not defined as an externally imposed training course or one-off workshop. Rather, it was conceptualized as involvement in a relatively complete cycle of structured music club activities characterized by continuity, collaboration, expression, and social visibility. To improve comparability across clubs, only activity cycles meeting the following criteria were included: (1) activities extended across at least 6 weeks; (2) activities involved collaborative musical practice rather than isolated attendance; (3) members had opportunities for some form of presentation, contribution, or expression; (4) activities were coordinated by stable organizers, student leaders, or instructors; and (5) participation required active engagement rather than passive observation alone.

Within this framework, the shared structure of music club participation was understood as comprising four interrelated practice domains. First, basic training and skill participation included vocal warm-ups, instrumental rehearsal, rhythm practice, section work, and technical preparation. Although often perceived as skill-oriented, such activities also functioned as initial sites through which members became present and recognizable within the group. Second, collaborative rehearsal and collective task completion involved ensemble coordination, group-based practice, arrangement discussion, and performance preparation. These activities were central to the development of mutual listening, interdependence, and group-based belonging. Third, opportunities for expression and presentation included internal sharing, rehearsal-based feedback, partial performance display, trial runs, and preparation for public or semi-public events. These moments potentially enabled members to move from simple attendance to more visible participation and voice. Fourth, post-activity feedback and informal interaction included organizer comments, peer encouragement, after-rehearsal conversations, and online group communication. Although often overlooked, these interactions may play an important role in shaping members’ sense of recognition, inclusion, and contribution.

Accordingly, music club participation in this study was treated as a relational and developmental context rather than a mere indicator of extracurricular interest, while recognizing that the study design cannot determine whether participation itself caused the observed changes.

### 3.4 Data collection procedures

Data collection was carried out across an 8-week activity cycle and involved three broad sources: quantitative survey data, lightweight student experience materials, and club process materials. To minimize disruption to routine club functioning, all data collection procedures were designed to be low-burden and embedded into ordinary activity rhythms as far as possible. The quantitative component consisted of two waves of questionnaire administration: T1 (pretest), administered 3–7 days before the beginning of the activity cycle, and T2 (posttest), administered within 1 week after the completion of the 8-week cycle. The questionnaire was primarily distributed online, with paper-based completion made available where necessary, and each wave required approximately 10–15 min to complete. To match pretest and posttest responses while preserving anonymity, participants generated their own non-identifiable personal code. No direct identifying information such as names or student numbers was collected on the survey instrument. The matched pretest-posttest survey constituted the core dataset used for the main statistical analyses and should be interpreted as supporting prospective associations over the activity cycle rather than strong causal inference.

To complement the survey data without imposing excessive demands on participants or organizers, four forms of lightweight supplementary material were collected. First, brief activity feedback forms were distributed at four key points in the activity cycle after the second week, fourth week, sixth week, and eighth week, and focused on perceived participation, comfort, inclusion, value of contribution, and overall atmosphere. A total of 248, 241, 236, and 229 feedback forms were collected across these four time points, resulting in 954 feedback entries overall, and 259 participants (89.9%) completed at least two feedback forms. Second, short student interviews were conducted after the posttest. A total of 74 students indicated willingness to participate, and purposive sampling was then used to ensure variation in club type, participation level, year group, and identity background. The research team ultimately interviewed 16 students, including 7 gender-marginalized participants. These interviews lasted between 10 and 19 min, with an average duration of 13.6 min, and focused on perceived changes in belonging, self-expression, and participation experience. Third, the research team conducted structured observations in seven of the nine clubs, observing two key activities in each selected club and generating 14 structured observation records in total. Observation settings included regular rehearsals, subgroup collaboration sessions, internal sharing activities, and performance-preparation meetings, and a brief observation template was used to record participation distribution, opportunities for voice, organizer facilitation, and peer response. Fourth, organizer activity logs were collected from 11 club organizers or core student leaders. Across the 8-week cycle, 72 organizer logs were planned and 61 valid logs were ultimately collected, representing a completion rate of 84.7%. Taken together, these supplementary materials provided modest contextual evidence for interpreting the quantitative findings and identifying interactional conditions that may have accompanied changes in belonging, identity development, and agency; they were not intended to constitute a fully independent qualitative analysis.

### 3.5 Measures

The questionnaire included five focal constructs: belonging, identity development, agency, perceived recognition, and expressive safety. Belonging referred to students’ perceived sense of acceptance, inclusion, and legitimate membership within the music club community. Identity development captured students’ perceived clarity, affirmation, and ability to express a meaningful sense of self through participation in music club activities. Agency referred to students’ perceived capacity to speak, contribute, influence group activities, and take initiative within the music club context. Perceived recognition reflected the extent to which students felt that their presence and contributions were noticed, valued, and acknowledged by others in the music club environment. Expressive safety referred to students’ sense of comfort and security in expressing themselves, sharing ideas, or participating visibly without fear of dismissal or negative judgment. All measures were adapted from established instruments in educational and developmental research and were contextually revised to fit the setting of university music club participation. Specifically, wording originally referring to school or classroom settings was modified to reflect students’ experiences in club-based musical practice, collaboration, and performance preparation. All focal items were rated on a five-point Likert scale ranging from 1 (strongly disagree) to 5 (strongly agree). Control variables were measured using categorical or continuous response formats as appropriate. Table 3 summarizes the adapted sources, number of items, and representative items of the key variables.

**Table 3:** Summary of key measures.

Construct	Adapted from	Number of items	Sample item
Belonging (BEL)	Adapted from Goodenow’s (1993) Psychological Sense of School Membership scale and related group/classroom belonging measures	5	“I feel that I am a real part of this music club.”
Identity Development (ID)	Adapted from established measures of identity expression, authenticity, and school-based self-concept development	5	“Participating in this music club helps me express who I am.”
Agency (AG)	Adapted from established measures of student agency, classroom voice, and participatory engagement	5	“I feel that my ideas can make a difference in this music club.”
Perceived Recognition (PR)	Adapted from recognition-related and school/community affirmation measures	4	“My contributions in this music club are recognized by others.”
Expressive Safety (ES)	Adapted from psychologically safe participation and expressive climate measures	4	“I feel safe expressing myself in this music club.”
Control variables	Developed based on prior research design conventions	—	Age, gender, year level, disciplinary background, duration of club participation, weekly participation frequency, and prior formal musical experience

## 4 Empirical approach

### 4.1 Descriptive statistics and correlations

Table 4 reports the descriptive statistics and bivariate correlations among the principal study variables. Overall, participants reported moderately positive levels of belonging (M = 3.70, SD = 0.65), identity development (M = 3.64, SD = 0.67), agency (M = 3.55, SD = 0.70), perceived recognition (M = 3.58, SD = 0.67), and expressive safety (M = 3.57, SD = 0.70), indicating that students generally evaluated their music club experiences positively while still showing sufficient variability for subsequent analysis. In terms of background characteristics, duration of club participation was positively associated with belonging (*r* = 0.34, *p* < 0.001), identity development (*r* = 0.25, *p* < 0.001), agency (*r* = 0.13, *p* < 0.05), perceived recognition (*r* = 0.27, *p* < 0.001), and expressive safety (*r* = 0.15, *p* < 0.01), suggesting that students with longer involvement in music clubs tended to report more favorable relational and developmental experiences. Weekly participation frequency showed a similar but generally stronger pattern, being positively related to belonging (*r* = 0.50, *p* < 0.001), identity development (*r* = 0.40, *p* < 0.001), agency (*r* = 0.41, *p* < 0.001), perceived recognition (*r* = 0.20, *p* < 0.001), and expressive safety (*r* = 0.25, *p* < 0.001), which indicates that more frequent engagement in club activities was associated with stronger perceived inclusion, self-development, and participatory confidence. With regard to the focal constructs, belonging was positively correlated with identity development (*r* = 0.59, *p* < 0.001), agency (*r* = 0.41, *p* < 0.001), perceived recognition (*r* = 0.37, *p* < 0.001), and expressive safety (*r* = 0.38, *p* < 0.001), while identity development was positively associated with agency (*r* = 0.29, *p* < 0.001), perceived recognition (*r* = 0.32, *p* < 0.001), and expressive safety (*r* = 0.50, *p* < 0.001). Agency was also positively related to perceived recognition (*r* = 0.20, *p* < 0.001) and expressive safety (*r* = 0.22, *p* < 0.001).

**Table 4:** Descriptive statistics and correlations among study variable.

Variable	M	SD	1	2	3	4	5	6	7
Age	20.20	1.24	—						
Duration of club participation	2.55	1.09	0.11†	—					
Weekly participation frequency	1.93	0.75	−0.05	−0.04	—				
Belonging (BEL)	3.70	0.65	0.02	0.34***	0.50***	—			
Identity development (ID)	3.64	0.67	−0.03	0.25***	0.40***	0.59***	—		
Agency (AG)	3.55	0.70	0.04	0.13*	0.41***	0.41***	0.29***	—	
Perceived recognition (PR)	3.58	0.67	0.07	0.27***	0.20***	0.37***	0.32***	0.20***	—
Expressive safety (ES)	3.57	0.70	−0.11†	0.15**	0.25***	0.38***	0.50***	0.22***	0.11†

^†^*p* < 0.10, *p < 0.05, **p < 0.01, ***p < 0.001.

### 4.2 Reliability and validity of the measures

The reliability and validity of the measurement model were examined prior to hypothesis testing. As shown in Table 5, all constructs demonstrated adequate to high internal consistency, with Cronbach’s alpha values ranging from 0.884 to 0.922, exceeding the commonly accepted threshold of 0.70. Although several alpha coefficients were relatively high, this pattern is not unexpected in the present study, given that the adapted items were intentionally focused on closely related experiences within a specific and bounded context, namely university music club participation. In other words, the relatively high internal consistency likely reflects the conceptual coherence of the measures in this setting rather than necessarily indicating problematic redundancy. At the same time, these coefficients should be interpreted with some caution, as very high internal consistency may also suggest that some items capture highly overlapping aspects of the same construct. Composite reliability (CR) values ranged from 0.884 to 0.914, likewise indicating satisfactory reliability across all latent variables. Convergent validity was also supported, as the average variance extracted (AVE) values ranged from 0.652 to 0.702, all well above the recommended minimum of 0.50, suggesting that each construct explained a substantial proportion of variance in its indicators. With respect to discriminant validity, the Fornell–Larcker criterion was satisfied: the square roots of the AVE values, shown on the diagonal of Table 5, ranged from 0.807 to 0.838 and were greater than the corresponding inter-construct correlations in the same rows and columns. This indicates that each construct shared more variance with its own indicators than with other constructs in the model.

**Table 5:** Reliability, convergent validity, and Fornell–Larcker criterion.

Construct	α	CR	AVE	BEL	ID	AG	PR	ES
BEL	0.903	0.903	0.652	0.807				
ID	0.914	0.914	0.680	0.593	0.825			
AG	0.922	0.922	0.702	0.408	0.288	0.838		
PR	0.884	0.884	0.655	0.374	0.324	0.204	0.810	
ES	0.894	0.895	0.680	0.376	0.497	0.216	0.111	0.824

Diagonal elements are the square roots of AVE.

In addition, Table 6 reports the HTMT ratios, all of which were below the conservative threshold of 0.85, providing further support for discriminant validity. Taken together, these results indicate that the measures used in the present study exhibited adequate reliability, satisfactory convergent validity, and acceptable discriminant validity, thereby supporting the suitability of the measurement model for the subsequent regression and mediation analyses.

**Table 6:** HTMT ratios for discriminant validity.

Construct	BEL	ID	AG	PR	ES
BEL	—				
ID	0.653	—			
AG	0.449	0.314	—		
PR	0.420	0.361	0.225	—	
ES	0.418	0.550	0.237	0.125	—

All HTMT values were below the conservative threshold of 0.85.

### 4.3 Pretest–posttest changes across the activity cycle

Table 7 presents the results of the paired-samples t-tests comparing pretest and posttest scores across the 8-week activity cycle. As shown, all five key variables increased significantly over time. Specifically, belonging rose from 3.56 (SD = 0.69) at pretest to 3.70 (SD = 0.65) at posttest, with a mean difference of 0.15, *t* = 6.965, *p* < 0.001, and a Cohen’s d of 0.41. Identity development increased from 3.48 (SD = 0.71) to 3.64 (SD = 0.67), yielding a mean difference of 0.16, *t* = 6.708, *p* < 0.001, *d* = 0.40. Agency likewise improved from 3.37 (SD = 0.73) to 3.55 (SD = 0.70), with a mean difference of 0.17, *t* = 6.997, *p* < 0.001, *d* = 0.41. In addition, perceived recognition increased from 3.43 (SD = 0.70) to 3.58 (SD = 0.67), *t* = 6.303, *p* < 0.001, *d* = 0.37, while expressive safety rose from 3.41 (SD = 0.73) to 3.57 (SD = 0.70), *t* = 6.650, *p* < 0.001, *d* = 0.39. Taken together, these findings indicate that participation across the activity cycle was associated with statistically significant positive changes in students’ relational, developmental, and participatory experiences. Although the magnitude of change was not large, the effect sizes were consistently in the small-to-moderate range, suggesting a meaningful but realistic pattern of positive change over time. Because the study did not include random assignment or a nonparticipating comparison group, these changes should be interpreted as associations observed across the activity cycle rather than as definitive effects caused by club participation.

**Table 7:** Paired-samples *t*-tests for pretest–posttest changes in key variables.

Variable	Pretest M (SD)	Posttest M (SD)	Mean difference	*t*	*p*	Cohen’s d
Belonging (BEL)	3.56 (0.69)	3.70 (0.65)	0.15	6.965	<0.001	0.41
Identity development (ID)	3.48 (0.71)	3.64 (0.67)	0.16	6.708	<0.001	0.40
Agency (AG)	3.37 (0.73)	3.55 (0.70)	0.17	6.997	<0.001	0.41
Perceived recognition (PR)	3.43 (0.70)	3.58 (0.67)	0.16	6.303	<0.001	0.37
Expressive safety (ES)	3.41 (0.73)	3.57 (0.70)	0.16	6.650	<0.001	0.39

### 4.4 Regression analyses of belonging, identity development, and agency

Table 8 presents the hierarchical regression results for belonging. In Model 1, the background variables explained a substantial proportion of variance in belonging (*R*^2^ = 0.416, *F* = 40.15, *p* < 0.001). Among these predictors, duration of club participation (*β* = 0.356, *p* < 0.001), weekly participation frequency (*β* = 0.527, *p* < 0.001), and prior formal musical experience (*β* = 0.200, *p* < 0.001) were all significant positive predictors, whereas age and gender were not significant. This suggests that students who had participated in music clubs for longer periods, engaged more frequently in club activities, and possessed greater prior musical experience tended to report stronger belonging. In Model 2, perceived recognition and expressive safety were added, and the explained variance increased to *R*^2^ = 0.474. Both perceived recognition (*β* = 0.166, *p* < 0.001) and expressive safety (*β* = 0.197, *p* < 0.001) emerged as significant positive predictors, while the coefficients for duration of participation, participation frequency, and prior musical experience remained significant but were reduced in magnitude, indicating that part of the effect of activity involvement on belonging may operate through students’ relational and expressive experiences within the club context. In Model 3, identity development and agency were further entered, increasing the explained variance to *R*^2^ = 0.542. In the final model, duration of club participation (*β* = 0.217, *p* < 0.001), weekly participation frequency (*β* = 0.307, *p* < 0.001), prior formal musical experience (*β* = 0.132, *p* < 0.01), perceived recognition (*β* = 0.105, *p* < 0.05), identity development (*β* = 0.295, *p* < 0.001), and agency (*β* = 0.131, *p* < 0.01) remained significant, whereas expressive safety was no longer significant.

**Table 8:** Hierarchical regression predicting belonging.

Variables	Model 1 β	Model 2 β	Model 3 β
Age	0.004	0.019	0.014
Women dummy	0.014	−0.005	0.008
Duration of club participation	0.356***	0.275***	0.217***
Weekly participation frequency	0.527***	0.438***	0.307***
Prior formal musical experience	0.200***	0.158***	0.132**
Perceived recognition (PR)	0.166***	0.105*	
Expressive safety (ES)	0.197***	0.071	
Identity development (ID)	0.295***		
Agency (AG)	0.131**		
R^2^	0.416	0.474	0.542
F	40.15***	36.08***	36.58***

Standardized coefficients are reported. *p* < 0.05, **p < 0.01, ***p < 0.001.

To further examine variables associated with identity development and agency, Table 9 reports regression analyses predicting these two outcomes. For identity development, the overall model was significant (*R*^2^ = 0.469, *F* = 30.80, *p* < 0.001). Weekly participation frequency (*β* = 0.130, *p* < 0.05), perceived recognition (*β* = 0.114, *p* < 0.05), expressive safety (*β* = 0.317, *p* < 0.001), and belonging (*β* = 0.341, *p* < 0.001) all emerged as significant positive predictors, whereas age, gender, duration of club participation, and prior formal musical experience were not significant. Among these predictors, belonging and expressive safety showed the strongest associations, suggesting that students were more likely to report stronger identity development when they felt securely able to express themselves and experienced a stronger sense of membership within the music club. By contrast, the model predicting agency was significant but explained a more modest proportion of variance (*R*^2^ = 0.232, *F* = 10.56, *p* < 0.001). In this model, weekly participation frequency (*β* = 0.280, *p* < 0.001) and belonging (*β* = 0.217, *p* < 0.01) were the only significant predictors, while perceived recognition, expressive safety, duration of participation, prior musical experience, age, and gender were not significant. This pattern indicates that agency was more directly associated with active and regular engagement in club activities, as well as with students’ sense of belonging, rather than with the full range of relational-process variables. Overall, the results in Table 9 suggest that identity development was most strongly associated with expressive safety and belonging, whereas agency was more closely tied to participation frequency and felt group membership.

**Table 9:** Regression analyses predicting identity development and agency.

Variables	Identity development β	Agency β
Age	−0.013	0.052
Women dummy	−0.050	0.016
Duration of club participation	0.061	0.039
Weekly participation frequency	0.130*	0.280***
Prior formal musical experience	0.026	−0.015
Perceived recognition (PR)	0.114*	0.050
Expressive safety (ES)	0.317***	0.057
Belonging (BEL)	0.341***	0.217**
R^2^	0.469	0.232
F	30.80***	10.56***

Standardized coefficients are reported. *p* < 0.05, **p < 0.01, ***p < 0.001.

### 4.5 Indirect effects of participation through recognition and expressive safety

To further examine possible indirect association patterns, Table 10 reports the bootstrapped indirect effects of weekly participation frequency on belonging, identity development, and agency via perceived recognition and expressive safety. As shown, all six indirect effects were statistically significant because their bias-corrected 95% confidence intervals did not include zero. For belonging, weekly participation frequency showed significant indirect associations through both perceived recognition (effect = 0.049, 95% CI [0.019, 0.085]) and expressive safety (effect = 0.058, 95% CI [0.028, 0.098]), suggesting that students who participated more frequently also tended to feel more recognized and safer in expressing themselves, and these conditions were associated with stronger belonging. A similar pattern emerged for identity development. Participation frequency showed a significant indirect association through perceived recognition (effect = 0.044, 95% CI [0.016, 0.082]) and an even stronger indirect association through expressive safety (effect = 0.094, 95% CI [0.050, 0.148]). This pattern suggests that expressive safety may be especially relevant to the link between regular participation and students’ identity-related development within the music club context. For agency, the indirect associations were smaller in magnitude but still significant, both through perceived recognition (effect = 0.024, 95% CI [0.003, 0.052]) and expressive safety (effect = 0.029, 95% CI [0.005, 0.063]). Taken together, these findings provide preliminary evidence of possible relational pathways linking participation frequency with belonging, identity development, and agency. However, because the study design does not fully establish temporal ordering among the mediators and outcomes, these mediation results should be interpreted as suggestive rather than confirmatory evidence of developmental mechanisms.

**Table 10:** Bootstrapped indirect effects of participation frequency on key outcomes via perceived recognition and expressive safety.

Indirect path	Effect	Boot SE	95% CI Lower	95% CI Upper
Frequency → PR → BEL	0.049	0.017	0.019	0.085
Frequency → ES → BEL	0.058	0.018	0.028	0.098
Frequency → PR → ID	0.044	0.017	0.016	0.082
Frequency → ES → ID	0.094	0.025	0.050	0.148
Frequency → PR → AG	0.024	0.013	0.003	0.052
Frequency → ES → AG	0.029	0.015	0.005	0.063

Bias-corrected bootstrap confidence intervals were based on 5,000 resamples.

### 4.6 Exploratory supplementary analyses for gender-marginalized students

Table 11 presents exploratory supplementary analyses focusing specifically on gender-marginalized students. As shown, the mean scores of gender-marginalized students were consistently lower than those of other participants across all five focal variables, including belonging, identity development, agency, perceived recognition, and expressive safety. Specifically, gender-marginalized students reported lower belonging (M = 3.58, SD = 0.73) than other participants (M = 3.72, SD = 0.64), lower identity development (M = 3.52, SD = 0.72 vs. M = 3.65, SD = 0.66), lower agency (M = 3.39, SD = 0.84 vs. M = 3.56, SD = 0.69), lower perceived recognition (M = 3.46, SD = 0.78 vs. M = 3.59, SD = 0.66), and lower expressive safety (M = 3.39, SD = 0.77 vs. M = 3.59, SD = 0.69). However, none of these between-group differences reached conventional levels of statistical significance. These results therefore should not be read as evidence of reliable subgroup differences. Rather, the consistent direction of the mean differences points to a possible pattern that warrants further investigation in studies with larger gender-marginalized samples. In the present study, the subgroup findings are best treated as preliminary and descriptive.

**Table 11:** Mean comparisons between gender-marginalized students and other participants.

Variable	Gender-marginalized students M (SD)	Other participants M (SD)	*t*	*p*
Belonging	3.58 (0.73)	3.72 (0.64)	−0.947	0.351
Identity development	3.52 (0.72)	3.65 (0.66)	−0.939	0.355
Agency	3.39 (0.84)	3.56 (0.69)	−1.014	0.319
Perceived recognition	3.46 (0.78)	3.59 (0.66)	−0.847	0.404
Expressive safety	3.39 (0.77)	3.59 (0.69)	−1.268	0.214

## 5 Discussion

### 5.1 Music club participation as a relational context for belonging, identity development, and agency

The first major finding of the present study is that participation in university music club activities was associated with a broadly positive pattern of change across the focal relational and developmental variables. As the pretest-posttest results showed, students reported significantly higher levels of belonging, identity development, agency, perceived recognition, and expressive safety after the 8-week activity cycle, with effect sizes in the small-to-moderate range. This pattern suggests that music club participation was linked not simply to change in one isolated domain, but to a more general strengthening of students’ relational, expressive, and participatory experiences. At the same time, the magnitude of change remained moderate rather than dramatic, which makes the pattern plausible in a natural educational context based on voluntary participation rather than tightly controlled intervention. The correlational and regression findings further reinforce this cautious interpretation. Greater involvement in club activities, particularly more frequent participation, was positively associated with belonging and with more favorable developmental outcomes, while perceived recognition and expressive safety were also positively related to the focal variables. Taken together, these results suggest that university music clubs may function as more than extracurricular leisure spaces. They appear to provide a relational setting in which repeated rehearsal, collaboration, and shared expression are associated with feeling more visible to others, more securely included within the group, and more able to participate with confidence. This interpretation is especially meaningful in relation to the present study’s exploratory attention to gender-marginalized students, for whom educational participation may be shaped not only by formal access, but also by whether the environment offers credible opportunities for recognition, inclusion, and voice. Although the present findings do not imply that music club participation by itself resolves broader structural forms of marginalization, they suggest that such settings may be associated with conditions under which more positive relational-developmental experiences become possible. Overall, this set of findings supports H1 and provides general support for H2 and H3, within the limits of the study design.

### 5.2 From recognition to development: the roles of belonging and expressive safety

A second contribution of the present study lies in identifying relational conditions that may help explain why music club participation is associated with student development. The findings suggest that belonging and expressive safety occupy particularly important positions in the association between participation and developmental outcomes, although their roles are not identical. For identity development, expressive safety and belonging emerged as the strongest predictors, while perceived recognition and participation frequency also made smaller but significant contributions. This pattern is theoretically meaningful, but it should be interpreted as evidence of association rather than proof of a causal mechanism. Identity development may require more than simple visibility or acknowledgement; it may depend on whether students feel sufficiently secure to express, test, and negotiate who they are within a shared social environment. In this sense, recognition may provide an initial signal that one is noticed, while expressive safety may provide a psychological condition under which self-expression can be sustained with less perceived risk. Belonging adds a further relational layer, because students are likely to explore and consolidate a more coherent sense of self when they feel that they are not merely present in the group, but genuinely part of it. Thus, the results suggest that identity development in music club settings may be embedded in the social experience of being accepted and of being able to express oneself without fear of dismissal or misreading. For this reason, the findings provide support for H4, while leaving the underlying developmental sequence for future research to test more directly.

The results for agency, however, reveal a somewhat different pattern, which is equally informative. Although agency was positively correlated with perceived recognition and expressive safety, the regression analyses showed that agency was more directly predicted by participation frequency and belonging. This suggests that agency may be less dependent on generalized feelings of recognition alone and more dependent on regular opportunities to enter, rehearse, and influence collective processes over time. In other words, feeling safe is important, but acting agentically may require repeated practice within the social and organizational life of the group. Students may become more agentic not simply because they feel affirmed, but because they participate often enough to see themselves as capable contributors whose actions matter within the collective setting. Belonging is also central here, because students are more likely to initiate ideas, speak up, and shape group interaction when they experience themselves as legitimate members rather than provisional participants. The indirect effects analysis reinforces this broader interpretation. Participation frequency was linked to belonging, identity development, and agency partly through perceived recognition and expressive safety, with expressive safety showing a particularly salient role for identity development. Taken together, these findings suggest that the developmental significance of music club participation lies not only in activity exposure itself, but in the relational and expressive conditions through which participation is experienced. In this sense, the present results support H5 and H6, while also giving more general support to the broader theoretical proposition that belonging, identity development, and agency are best understood as interconnected dimensions rather than isolated outcomes.

### 5.3 Implications for gender-marginalized students, educational practice, and future research

The supplementary analyses concerning gender-marginalized students add an important but exploratory interpretive layer to the present findings. Although the between-group comparisons did not reach conventional levels of statistical significance, the mean scores of gender-marginalized students were consistently lower than those of other participants across belonging, identity development, agency, perceived recognition, and expressive safety. This directional consistency is noteworthy, but the subgroup size limits statistical power and prevents strong conclusions about between-group differences. The findings therefore suggest a possible pattern rather than a confirmed subgroup effect: gender-marginalized students may experience somewhat less favorable relational and developmental conditions, even within contexts that are, on the whole, supportive. From this perspective, the present study does not show that music clubs automatically eliminate inequality; rather, it suggests that such settings may still reflect broader asymmetries in recognition, comfort, and participation, even as they provide positive developmental possibilities for many participants. This is precisely why the present findings should be interpreted as cautious and preliminary: they point to the importance of studying gender-marginalized students in music-based educational settings without overstating what the current subgroup evidence can establish.

These findings also carry practical implications for educators, club organizers, and institutions. Most importantly, the results suggest that the value of music-based educational participation does not lie simply in offering artistic activities, but in how these activities are socially organized. If belonging, identity development, and agency are associated with recognition and expressive safety, then the crucial practical question is not only whether students participate in music, but whether the environment is structured so that they can be seen, heard, and included as legitimate contributors. This means that supportive music education should not rely only on general encouragement or symbolic inclusion. It should instead create repeated and credible opportunities for contribution, reduce the interpersonal risks associated with self-expression, and ensure that feedback, rehearsal practices, and group routines do not silently reproduce hierarchies of visibility and legitimacy. At the same time, these implications should be read as practice-oriented interpretations rather than direct evidence of intervention effects. Future research could extend this work by examining larger gender-marginalized samples, comparing formal classroom and co-curricular music settings, and testing more elaborated developmental pathways across longer periods of participation.

## 6 Conclusion

This study examined whether participation in university music club activities was associated with students’ belonging, identity development, and agency, with exploratory attention to gender-marginalized participants. Across the 8-week activity cycle, students reported significant increases in belonging, identity development, agency, perceived recognition, and expressive safety, and the subsequent regression and indirect-effect analyses suggested that these changes were linked to relational and expressive conditions within club participation. The findings indicate that music club participation may matter because it is associated with contexts in which students feel more recognized, more securely included, and more able to act within a shared community. In this sense, the present study supports the view that university music clubs can function as educationally meaningful environments rather than as purely recreational extracurricular spaces. More specifically, the results suggest that belonging occupies a foundational role in the broader developmental pattern, while expressive safety appears especially relevant to identity development and frequent participation appears especially relevant to agency. These patterns clarify that the value of music-based participation lies not only in artistic engagement itself, but also in the way such participation structures opportunities to be seen, heard, and included. For students whose participation in mainstream educational life may otherwise be shaped by invisibility, misrecognition, or conditional legitimacy, this kind of environment may provide a meaningful context for more positive developmental experience. Although the supplementary analyses for gender-marginalized students should be interpreted cautiously, the consistently lower means across the focal variables suggest a possible descriptive pattern that warrants further study. Taken together, the present study makes three main contributions. First, it extends research on gender-marginalized students by examining a co-curricular music setting that has been comparatively neglected in the literature. Second, it offers a relational-developmental perspective in which belonging, identity development, and agency are treated as theoretically connected rather than isolated outcomes. Third, it provides pretest-posttest evidence that structured participation in music club activities is associated with positive educational change over time and with recognition- and safety-related relational conditions. Overall, the findings suggest that music-based educational communities may offer an important, if modest, context through which students experience stronger membership, clearer self-development, and more active participation.

## 7 Limitations

This study has several limitations. First, although the pretest-posttest design provides stronger evidence than a purely cross-sectional design, the absence of random assignment, a nonparticipating comparison group, or longer-term follow-up means that causal claims should remain cautious. The findings show positive changes across an 8-week activity cycle, but they cannot establish that music club participation alone caused those changes. Second, the mediation analyses should be interpreted as suggestive rather than confirmatory. Because the study did not fully separate participation, mediators, and outcomes across multiple time points, the analyses cannot decisively establish temporal ordering or prove a developmental mechanism. Third, the subgroup of gender-marginalized participants was relatively small, which constrained statistical power and requires cautious interpretation of subgroup-related findings. Fourth, although the study used supplementary interviews, feedback forms, observations, and organizer logs, these materials served mainly as contextual evidence; they did not constitute a fully developed qualitative strand. Fifth, because the study was conducted in university music club settings rather than formal music classrooms, the generalizability of the findings to other educational contexts may be limited. Finally, the study focused on a limited set of relational and developmental variables, and other contextual influences such as institutional climate, peer norms, prior experiences of marginalization, family support, or broader campus policies were not directly examined. Future research should employ longer-term longitudinal designs, more rigorous comparison groups, larger gender-marginalized samples, and fuller mixed-methods integration to clarify the temporal ordering, subgroup dynamics, and contextual conditions underlying these developmental patterns.

## Data Availability

The raw data supporting the conclusions of this article will be made available by the authors, without undue reservation.
