# Correction: University music club participation, belonging, identity development, and agency: an exploratory pretest-posttest study with attention to gender-marginalized students

**DOI:** 10.3389/fpsyg.2026.1900264

**Published:** 2026-06-30

**Authors:** ZiLong Guo, Anqian Wang, Keran Li, Gao Qi, Yaojue Xie

**Affiliations:** 1 Public Basic Department, Zhejiang Police College, Hangzhou, China; 2 Faculty of Educational and Liberal Studies, City University, Petaling Jaya, Selangor, Malaysia; 3 Faculty of Music and Dance, Liaocheng University, Liaocheng, China; 4 Global Convergence Studies Department, Kangwon National University, Chuncheon-si, Republic of Korea; 5 Music Academy Department, Dankook University, Yongin-si, Republic of Korea

**Keywords:** agency, belonging, expressive safety, gender-marginalized students, identity development, music education, recognition, university music clubs

Author ZiLong Guo was erroneously assigned to affiliation Faculty of Music and Dance, Liaocheng University, Liaocheng, China. This affiliation has now been removed for author ZiLong Guo.

Affiliation Public Basic Department, Zhejiang Police College, Hangzhou, China was omitted from the paper. This affiliation has now been added for author ZiLong Guo.

Author Keran Li was erroneously assigned to affiliation Global Convergence Studies Department, Kangwon National University, Chuncheon-si, Republic of Korea. This affiliation has now been removed for author Keran Li.

Affiliation Faculty of Music and Dance, Liaocheng University, Liaocheng, China was omitted for author Keran Li. This affiliation has now been added for author Keran Li.

Author Gao Qi was erroneously assigned to affiliation Music Academy Department, Dankook University, Yongin-si, Republic of Korea. This affiliation has now been removed for author Gao Qi.

Affiliation Global Convergence Studies Department, Kangwon National University, Chuncheon-si, Republic of Korea was omitted for author Gao Qi. This affiliation has now been added for author Gao Qi.

The original version of this article has been updated.

